# A millennium of north-east Atlantic cod juvenile growth trajectories inferred from archaeological otoliths

**DOI:** 10.1371/journal.pone.0187134

**Published:** 2017-10-27

**Authors:** Guðbjörg Ásta Ólafsdóttir, Gróa Pétursdóttir, Hlynur Bárðarson, Ragnar Edvardsson

**Affiliations:** 1 University of Iceland, Research Centre of the Westfjords, Adalstraeti Bolungarvik, Iceland; 2 Marine and Freshwater Research Institute, Skúlagata Reykjavik, Iceland; Universita degli Studi di Bari Aldo Moro, ITALY

## Abstract

Archaeological excavations of historical fishing sites across the North Atlantic have recovered high quantities of Atlantic cod (*Gadus morhua*) bones. In the current study we use Atlantic cod otoliths from archaeological excavations of a historical fishing sites in north-west Iceland, dated to AD 970 –AD 1910 to examine historical growth trajectories of cod. No large scale growth variations or shifts in growth patterns were observed in the current chronologies, supporting the stability of historical Atlantic cod growth trajectories. The most significant variation in growth patterns was consistent with those that have been observed in recent times, for example, reduced early juvenile growth during periods of colder ocean temperature. The current results represent a high resolution chronological record of north-east Atlantic cod growth, greatly increasing the prior temporal range of such data, thereby providing a valuable baseline for a broad range of studies on Atlantic cod growth.

## Introduction

Historical baselines of ecological states can improve the interpretation of current anthropogenically induced change. Such baselines have already shown their value as a guide to modern day management and conservation [1]. Records of fish landings and imports have, for example, been used to reconstruct past fish abundance and size [2–7]. Zoo-archaeological material of exploited animal populations may be particularly useful for reconstructing historical baselines [8] as they provide a population level link between written historical sources and paleo-environmental data series.

Otoliths, a calcified structure in the inner ear of fish, are occasionally recovered during archaeological excavations [9]. They are unique among zoo-archaeological material in that they simultaneously convey information on individual fish age [10], growth [11] and reflect the environmental conditions that the fish encountered throughout life-history [12–14]. Otoliths from archaeological excavations have been used to examine historical changes in fish age [15, 16], trophic position [17] and seasonality of human site occupation [18]. Importantly, otoliths allow measures of annual growth, and thereby retrospective reconstructions of fish growth patterns. Growth reconstructions from archaeological otoliths have for example shown higher growth rate of Neolithic Baltic cod than in the modern population, particularly in the first year of growth [19]. Conversely, research from the North Sea finds slightly slower growth rate of cod (as well as haddock and plaice) in early modern times than in recent times [16].

Fish growth trajectories are plastic and correlate with a number of environmental factors including food availability [20], temperature [21, 22] and acidification [23]. Recent reductions in length at age have been noted for many exploited fish species [24–26]. The trend for reduced size is attributed to fisheries induced selection on fish life history traits, importantly on size at maturity, as fishing with common gear, such as trawls, favors individuals that mature at a smaller size [27–29]. Climate change may also result in evolutionary reductions in fish size through physiological adaptations [30]. To facilitate interpretation of ongoing change in fish growth long term historical time series of growth trajectories are needed.

Archaeological excavations of historical fishing sites across the North Atlantic have unearthed high quantities of Atlantic cod bones [31, 32] and the species composition of bone assemblages suggests an early specialization on Atlantic cod fisheries [33–35]. The quantity and often good preservation of the zoo-archaeological material at these sites offer unparalleled opportunities of retrospective examinations of Atlantic cod biology, including estimation of historical growth trajectories. Previous research on Atlantic cod bones from archaeological excavations have suggested ecological changes before industrial fisheries, for example, a disruption in growth of north-east Arctic cod [36] and loss of genetic diversity in Icelandic cod in the 16^th^ century [37].

In the North Atlantic, the medieval and early modern periods were characterized by rapid increase in marine fisheries; as urbanization and globalization in western Europe drove increasing demand for stock fish; and multinational fishing fleets sought favorable fishing grounds for Atlantic cod [38]. At the same time a cooling climate significantly affected societies across northern Europe, with the onset of the “little ice age” and subsequent temperature fluctuation; including a North Atlantic temperature minimum in the 17^th^ century [39, 40]. Adult Atlantic cod are tolerant to a wide range of temperature [41] and are known to migrate to areas with favorable temperature [41, 42]. However, age 0+ juveniles are dependent on shallow nearshore areas and may therefore be more affected by changes in sea temperature [43, 44].

In the current study, we analyze growth patterns of Atlantic cod otoliths from archaeological excavations of historical fishing sites in NW Iceland, dated to AD 970 –AD 1910. First, we examine the significance of change in otolith size, linear and quadric growth patterns across the millennium. Our initial hypothesis was based on faster growth in the medieval warm period followed by reductions in growth rate, particularly during the North Atlantic temperature minimum in the 17^th^ century.

## Materials and methods

### Archaeological excavations and zoo-archaeological analysis

**Fig 1:**
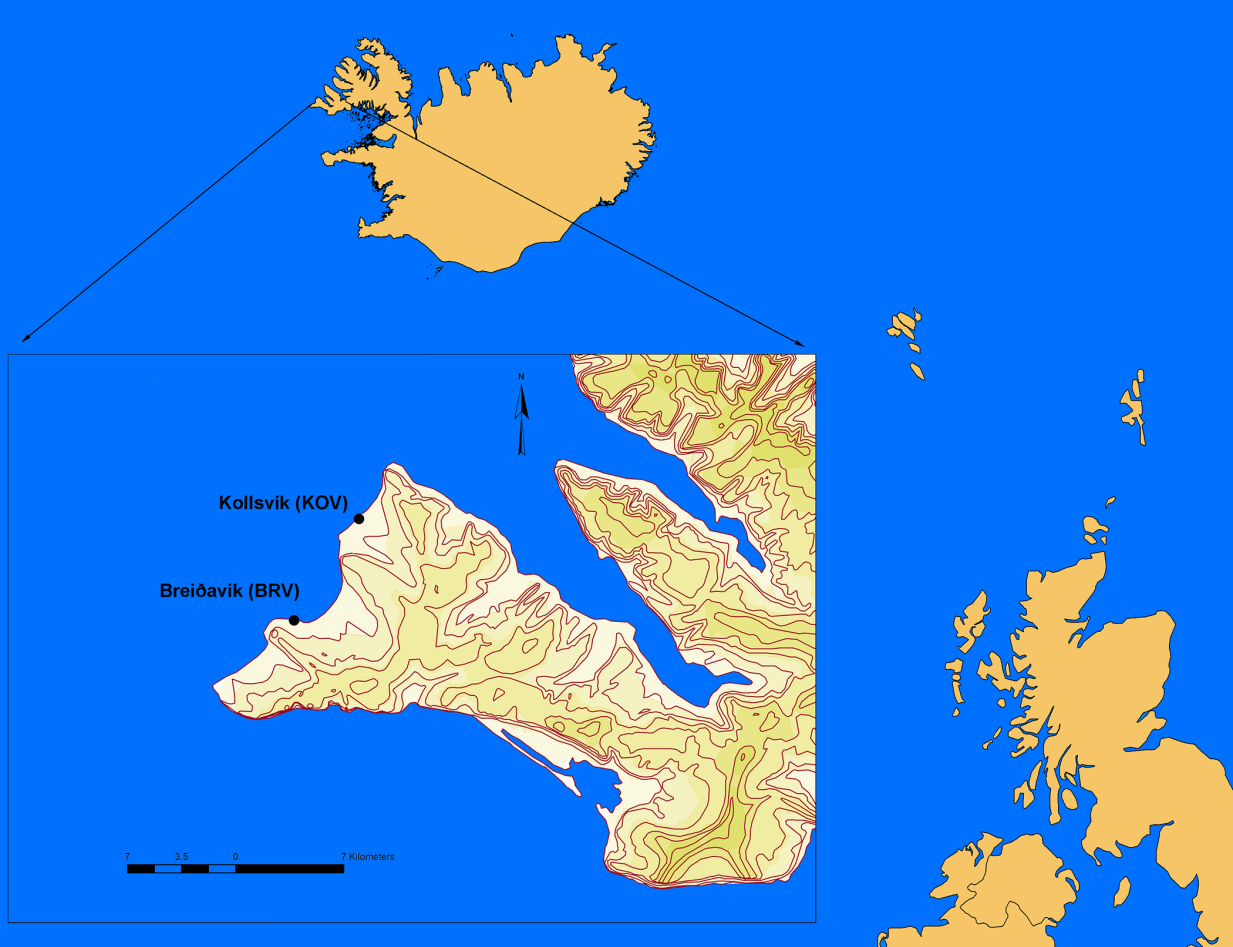
Map depicting sample sites. The map shows Breiðavík (BRV) and Kollsvík (KOV) the historical fishing sites were the excavations were conducted.

The archaeological excavations were carried out at the historical fishing sites; Breiðavík (BRV, 24°24'45.98”W, 65°32'38.13"N) and Kollsvík (KOV, 24°21´6.19”W, 65°36´36.07”N) in north-western Iceland (Fig 1). In July 2012 we excavated two trenches; one in Breiðavík (50cm x 50cm) and one in Kollsvík (1m x 80cm). In July 2015 we again excavated two trenches; both in Breiðavík (the first 2m x 50cm, the second 1m x 50cm).

Archaeological units, i.e. individual cultural deposits, were identified, recorded and excavated in reversed order, starting with the youngest. Each deposit was sieved with a 4mm mesh to retrieve bones, otoliths and finds. During the post excavation work all identifiable bones were identified to a species level and all Atlantic cod otoliths were removed from the bone assemblage for further analysis. The deposits were initially dated by their context, i.e. stratigraphical sequence or finds, and ultimately by ^14^C dating (Scottish Universities Environment Research Centre). Mean ^14^C age, quoted in years AD, was used in analysis. The error around the mean (Table 1), is expressed at the one sigma level of confidence, including components from the counting statistics on the sample, modern reference standard and blank and the random machine error. Five deposits could not be dated by ^14^C and were assigned an “informed mean date” based on their stratigraphical sequence and ^14^C dates of the adjacent deposits formed the error around the informed mean date (Table 1).

**Table 1:** Summary information of the otoliths used for age and growth analysis.

Deposit date	Dating	Site.year	n otoliths	Age mean	Age SD
AD 970	C14 (970±30)	BRV.15	17	10.18	3.45
AD 1360	Context (< 1410)	BRV.15	5	13.00	4.92
AD 1410	C14 (1410±30)	BRV.15	44	10.52	2.47
AD 1490	Context (1410–1570)	BRV.15	7	8.29	2.75
AD 1570	Context (1410–1570)	BRV.15	9	5.67	0.95
AD 1621	C14 (1621±30)	BRV.12	6	8.67	2.67
AD 1637	C14 (1637±32)	BRV.15	23	7.94	2.09
AD 1649	C14 (1649±32)	BRV.15	7	7.00	2.92
AD 1660	Context (1637–1680)	BRV.15	11	9.36	2.83
AD 1680	C14 (1680±32)	BRV.15	7	10.14	2.84
AD 1710	C14 (1710±28)	KOV.12	10	11.80	1.80
AD 1744	C14 (1744±32)	KOV.12	14	10.50	2.74
AD 1785	C14 (1785±30)	BRV.15	6	8.50	3.04
AD 1795	C14 (1795±28)	KOV.12	16	10.47	2.98
AD 1820	C14 (1820±32)	BRV.15	12	8.25	3.14
AD 1890	C14 (1890±32)	BRV.15	11	8.45	3.88
AD 1910	Context (>1890)	BRV.15	15	8.07	2.95

Sample sizes of otoliths from each archaeological deposit. Dating information is given as mean ^14^C dates and the associated error and for deposits with no ^14^C information as informed estimates and range (see text for details). Age represents fish otolith age within deposits.

We used a total of 220 archaeological specimens for the current analysis. Sample numbers can be found in S1 File. The archaeological excavations were permitted by the Icelandic Cultural Heritage Agency: Permit no: 21505–0060. The samples used in this study were deposited at the National Museum of Iceland: Conservation no: 2015–33.

### Otolith aging and annotation

A total of 220 otoliths were used for aging and growth determination, n = 57 from the 2012 excavation and n = 163 from the 2015 excavation (Table 1). All otoliths used were well preserved, although broken otoliths were used for age determination and growth estimates when it was possible to clearly identify all annual rings. Age determination and growth measures were done “blinded”, that is, without information on ^14^C dating.

**Fig 2:**
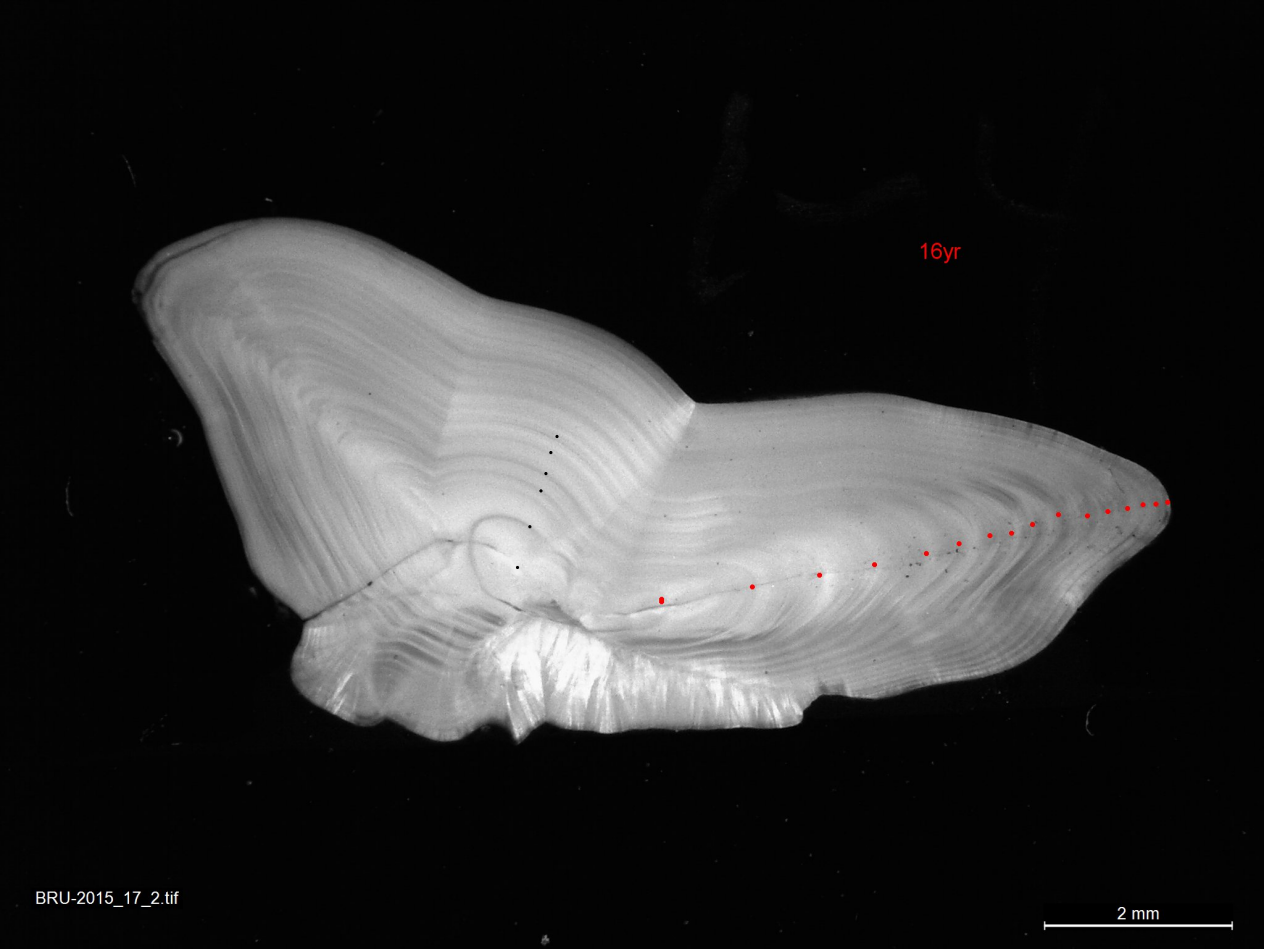
Otolith thin section from the transverse midplane. The red dots show age denotation and the black dots an example of five-year increment measures from the core to the outer distal edge of the otolith.

The sagittae otoliths were sectioned along the transverse axis (cross-sectioning), this involves embedding the otoliths in black resin blocks, removing a thin section from the transverse midplane. Images were taken of each otolith again using Leica IC80 HD (Digital Camera Module by Leica Microsystems) under a stereomicroscope at 10× magnification using a reflected light setting, with resolution 2048 x 1536 pixels. The age determination and annotation of cod otoliths in this study were carried out by highly experienced otolith reader. The otolith sizes at the mid-point of respective annual translucent zones were then marked along a transect from the core to the outer distal edge of the otolith, with the transect being approximately perpendicular to the width of the otolith (following Li et al. [11], Fig 2). Only the first five years of growth were measured. Fish growth slows with age and accurate estimates of annuli growth along the distal axis became increasing difficult with age, particularly in the very old individuals.

We did not attempt back calculations of fish size as archaeological otoliths are known to be subject to shrinkage and estimates of life fish length based on archaeological otoliths may be underestimated [9]. We therefore used otolith increment measures directly in subsequent statistical analysis. Note that this equates to not consider the biological intercept model [45], that is, the non-linear relationship between somatic and otolith growth. Both the data and figures presented should be interpreted with this in mind.

### Statistical analysis

First, we examined if growth patterns differed between archaeological deposits using the first five years of growth of 220 otoliths, a total of 1100 growth measures (Table 1). A second-order polynomial (Eq 1) was fitted using a generalized linear mixed model (GLMM) [46].

$$Y_{ij}=\beta_{0i}+\beta_{1i}\cdot Time_{j}+\beta_{2i}\cdot Time^{2}{}_{j}+\varepsilon_{ij}$$ (1)

Where Yij is the otolith size observation of individual i at Time j, *β*_0i_ is the intercept, *β*_1i_ is the linear slope and *β*_2i_ the quadratic curvature, ε_ij_ is the residual error and T is time of the observation (fish age in years). The mean estimated date (AD) for each archaeological deposit (Table 1) formed a fixed effect on both the linear and quadric term and fish ID and fish age at mortality were set as random effects. We used the deposit closest to modern times as the reference point (AD 1910). Statistical significance (p-values) for parameter estimates was assessed using the normal approximation (i.e., treating the t-value as a z-value). Models were fitted using the lme4 package v. 1.1–12 [47] in R version 3.0.2 [48].

Second, as there were often few otoliths in each archaeological deposit, we divided the otoliths into three non-overlapping temporal groups, the first represented samples dated to before AD 1499 (Medieval period), the second group included samples dated to AD 1500—AD 1784 (Early Modern period) and finally samples dated to post AD 1785 (Modern period). This classification represents a common historical designation of periods. We then repeated the GLMM, as described above, but replacing archaeological deposit with period as a fixed effect.

Finally, we estimated the typical von Bertalanffy growth function (vBGF, Eq 2) parameters for each of the three periods (Medieval, Early Modern and Modern).

$$Y_{ij}=L_{\infty }*1-exp\left(-K*\left(Time_{j}-t_{0}\right)\right)$$ (2)

Where Yij is the otolith size observation of individual i at Time j and T is time of the observation (fish age in years). *L*_∞_ is the maximum length (in the current analysis of otolith increment), *K* is the relative growth rate, *t*_0_ is the theoretical age for time at which length is zero. The vBGF is widely used in fisheries biology [49, 50] and our aim was to facilitate comparison of historical Atlantic cod growth rate to previous studies as well as to test for parameter differences between periods. Two models were fitted, 1) a model assuming the same parameters values for all 220 otoliths and 2) a model allowing all parameters to differ between the three periods. vBGF models 1 and 2 were then compared using a likelihood ratio test. Models were implemented in R version 3.0.2 [48] using the package FSA [51]. The current growth data is based on otolith increments and within individual measures are expected to be non-independent. Therefore, we attempted to fit a non-linear mixed model with a user defined vBGF using nlme in the lme4 package v. 1.1–12 [47]. However, vBGF models including random effects did not converge (data not shown). Code for statistical analysis can be found in S2 File.

## Results

**Fig 3:**
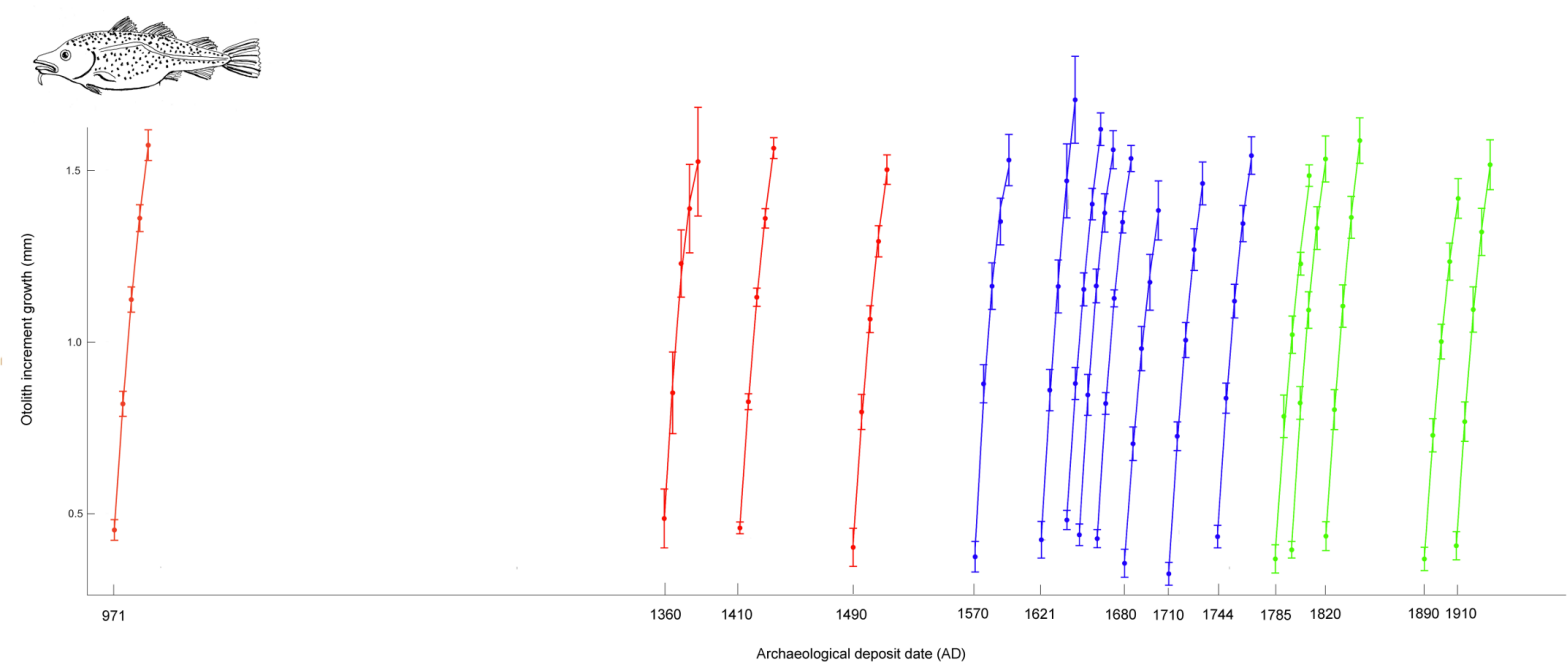
Raw otolith increment growth data and fitted growth patterns. The figure depicts five-year otolith increment growth (mean and SE), as well as the fitted quadric growth patterns for each of the seventeen archaeological deposits. Archaeological deposits are represented by mean ^14^C or estimated mean date AD. The color represents designated historical periods, red = Medieval, blue = Early modern and green = Modern.

The results support consistent growth patterns of Atlantic cod through the millennium, that is, repeated fluctuations but no long term shifts in growth patterns. Fig 3 depicts the otolith growth data and fitted growth patterns for each archaeological deposit examined.

In the first generalized linear mixed model, comparing across all archaeological deposits, the intercept, the linear term and the quadratic term were all significant (Table 2) showing that both linear and quadratic growth curves represent the observed growth pattern (note that this does not indicate any difference between archaeological deposits). The effects of archeological deposit as a fixed effect on otolith increment size was significant for AD 1680 (using AD 1910 as a base for comparison) (Table 2). There were significant interaction effects of the linear term and the archaeological deposits dated to AD 1680 and AD 1621, showing significantly lower linear growth rate in AD 1680 and higher linear growth rate in AD 1621. Finally, there was significant interaction of the quadric term the archeological deposit dated to AD 1570, representing increased quadric curvature, that is, relatively slower growth at age 3+ and age 4+.

**Table 2:** Results from the generalized linear mixed model analysis of growth patterns.

	Estimate	SE	t-value	p-value
Intercept	1.080	0.043	25.390	0.000
Linear term	0.878	0.022	40.644	0.000
Quadric term	-0.110	0.021	-5.285	0.000
AD 1680	-0.150	0.074	-2.025	0.043
Linear term: AD 1621	0.122	0.038	3.220	0.001
Linear term: AD 1680	-0.091	0.036	-2.512	0.012
Quadric term: AD 1570	-0.070	0.033	-2.113	0.035

Note that only significant result (p < 0.05) are presented. Full model results can be found in S1 Table.

When the otoliths were grouped by periods, the intercept, the linear term and the quadratic term were all significant but no significant difference in growth patterns were observed between periods (S2 Table).

Estimates of vBGF parameters did not differ significantly between periods, that is, the model allowing all vBGF parameters to differ between periods was not a significantly better fit than a model with the same parameter values for all otoliths (df = 6, difference in log likelihood = -0.61, χ^2^ = 1.23, p = 0.98). *L*_*∞*_ estimates for the three periods varied between 2.203 and 2.441, estimates of *K* varied between 0.204 and 0.248 and estimates of *t*_*0*_ varied between -0.018 and 0.124 (Table 3). Any comparison of these parameter values should acknowledge the shrinkage of archeological otoliths [9].

**Table 3:** Estimates and 95% confidence intervals of the vBGF model parameters, *L*_*∞*,_ *K* and *t*_*0*_, for the medieval, early modern and modern periods.

	Medieval (< AD 1499)			Early Modern (AD 1500—AD 1784)			Modern (> AD 1785)		
	*L*_*∞*_	*K*	*t*_*0*_	*L*_*∞*_	*K*	*t*_*0*_	*L*_*∞*_	*K*	*t*_*0*_
Estimate	2.343	0.219	0.020	2.441	0.204	-0.018	2.203	0.248	0.124
95% LCI	1.988	0.144	-0.220	1.915	0.078	-0.487	1.974	0.182	-0.044
95% UCI	2.993	0.308	0.222	4.563	0.331	0.279	2.608	0.312	0.271

## Discussion

Examination of growth trajectories across AD 970 to AD 1910 showed significantly slower growth, as well as smaller total otolith increment lengths, in the late 17^th^ century signaling reduced growth of age 0+ juveniles. Other notable changes in the polynomial growth model include negative estimates of the quadratic term in AD 1570, suggesting slower growth of age 3+ and age 4+ juveniles, and finally steeper linear growth in AD 1621 (Table 2, Fig 3). Despite these variations between the archaeological deposits we highlight that no consistent or long term shifts in Atlantic cod growth patterns growth trajectories were noted between periods, as may have been expected, for example, between the medieval warm period and modern times (Fig 3, S2 Table).

Atlantic cod growth has been examined across 20^th^ century time series that have shown considerable short term and inter-annual fluctuations in growth [52, 53]. Archaeological datasets do not capture intra-annual or between cohort variation as cohorts and multiple years are inevitable pooled within a single archaeological deposit. The current results may therefore underestimate temporal fluctuations in growth and this is further indicated by the loss of any significant effects when the otoliths were pooled to three historical periods (S2 Table). However, the current data signals a notable decline in juvenile growth the 17^th^ century. This is consistent with the results of Geffen et al., [36] that showed decreased growth of north-east Arctic cod between the early 16^th^ century and the 18^th^ century. The current results add to those previously reported as they provide a second geographically distinct dataset and the temporal resolution of the current data allows further deductions on the timing and extent of growth shifts in Atlantic cod.

The reduction in growth in the late 17^th^ century appears to represent slower growth of age 0+ juveniles (Fig 3). The North Atlantic cooled in the 17^th^ century and historical documents report harsh winters and inshore conditions e.g. icebergs and ice covered fjords around Iceland [39, 40]. Juvenile Atlantic cod nursery areas are in inshore waters and age 0+ juveniles are particularly likely to be found in shallow nearshore waters [43, 44]. Atlantic cod age 0+ juveniles may therefore be more effected by local climate effects than older cod that can seek favorable temperature and foraging conditions [41, 42]. Previous research has shown that ocean temperature was not a primary source of otolith growth variation in juvenile Atlantic cod [13]. However, lower sea temperatures in the 17^th^ century may also have affected food availability. Juvenile cod feed predominantly on zooplankton and are dependent on phenological matching of zooplankton blooms [54–56]. This matching may be disrupted by climate effects suggesting that food limitation could also explain slower growth of age 0+ juvenile Atlantic cod in the 17^th^ century.

The current data suggests that Atlantic cod growth was not more rapid in the warmer period preceding the 17^th^ century sea temperature minimum. In fact, growth in the 4^th^ and 5^th^ year of life was less rapid in AD 1570 (Table 2, Fig 3). This result may be consistent with previous research that show that adult growth is maximized at cooler sea temperatures [41, 57–59], as well as studies from the 20^th^ century that have shown that warmer periods in Icelandic waters negatively impact cod, primarily through northward migrations of capelin; favored forage fish [60]. Finally, the current growth reconstructions support that the large sized fish described in the medieval and early modern periods by anecdotes and archaeological reconstruction [1, 31] is not likely to represent a shift in growth patterns but the higher age of the pristine cod populations. Higher mean age of historical Atlantic cod populations has been found in previous studies [31, 37] and the current study (Table 1).

As any fisheries samples, archaeological fish remains can be biased, for example; by season, fishing methods and market preferences, all of which could affect the size of the landed fish. A particular consideration for interpreting growth patterns based on Atlantic cod otoliths from archaeological sites is that growth trajectories differ between populations of Atlantic cod, importantly, between migratory and coastal ecotypes [61, 62]. Any shifts in population distributions or in the frequency of populations or ecotypes in the catch could result in concurrent signals of change in growth patterns. Ólafsdóttir et al., [37] reported lower incident of *PanI* genotypes, representative of migratory Atlantic cod, in archaeological samples dated to post AD 1600. Therefore, we suggest that further research is needed to conclude on historical growth trajectories of migratory and coastal ecotypes.

To conclude, the current results provide a high resolution chronological record of consistent growth patterns of north-east Atlantic cod on a millennium scale; a potentially valuable baseline for modern day studies of environmental effects on Atlantic cod growth. We moreover propose that further study on otoliths from archaeological excavations has the potential to increase understanding on environmental effects on fish growth trajectories.

## Supporting information

S1 Table Full results from the first generalized linear mixed model. (DOCX)

S2 Table Results from the second generalized linear mixed model. (DOCX)

S1 File Supporting data including all data used in the analysis presented. (XLSX)

S2 File R code for the statistical models. (DOCX)

## References

[1] Engelhard GH, Thurstan RH, MacKenzie BR, Alleway HK, Bannister RCA, Cardinale M, et al Anecdotes and the shifting baseline syndrome of fisheries. Trends Ecol Evol. 1995; 10: 430 21237093 10.1016/s0169-5347(00)89171-5

[2] Jackson JB, Kirby MX, Berger WH, Bjorndal KA, Botsford LW, Bourque BJ, et al Historical overfishing and the recent collapse of coastal ecosystems. Science. 2001; 293: 629–637. doi: 10.1126/science.1059199 11474098 10.1126/science.1059199

[3] Pinnegar J, Engelhard GH. The ‘shifting baseline’phenomenon: a global perspective. Rev Fish Biol Fish. 2008; 18: 1–16.

[4] Poulsen B, Holm P, MacKenzie BR. A long-term (1667–1860) perspective on impacts of fishing and environmental variability on fisheries for herring, eel, and whitefish in the Limfjord, Denmark. Fish Res. 2007; 87: 181–195.

[5] MacKenzie BR, Bager M, Ojaveer H, Awebro K, Heino U, Holm P, et al Multi-decadal scale variability in the eastern Baltic cod fishery 1550–1860—evidence and causes. Fish Res. 2007; 87: 106–119.

[6] Opdal AF, Jørgensen C. Long‐term change in a behavioural trait: truncated spawning distribution and demography in Northeast Arctic cod. Global Change Biol. 2015; 21: 1521–1530. 10.1111/gcb.12773 PMC4404994 25336028

[7] Orton DC. Archaeology as a Tool for Understanding Past Marine Resource Use and Its Impact In: Schwerdtner Máñez K, Poulsen B, editors. Perspectives on Oceans Past. A handbook of Marine Environmental History. Springer; 2016 pp. 47–69.

[8] Disspain MC, Ulm S, Gillanders BM. Otoliths in archaeology: methods, applications and future prospects. J Archaeol S—R. 2016; 6: 623–632.

[9] Reibisch J. Ueber die Eizahl bei Pleuronectes platessa und die Altersbestimmung dieser Form aus den Otolithen. Wissenschaftliche Meeresuntersuchungen (Kiel). 1899; 4: 233–248.

[10] Li L, Høie H, Geffen AJ, Heegaard E, Skadal J, Folkvord A. Back-calculation of previous fish size using individually tagged and marked Atlantic cod (Gadus morhua). Can J Fish Aquat Sci. 2008; 65: 2496–2508.

[11] Campana SE, Casselman JM. Stock discrimination using otolith shape analysis. Can J Fish Aquat Sci. 1993; 50: 1062–1083.

[12] Campana SE. Year-class strength and growth rate in young Atlantic cod Gadus morhua. Mar Ecol Prog Ser. 1996; 135: 21–26.

[13] Campana SE, Thorrold SR. Otoliths, increments, and elements: keys to a comprehensive understanding of fish populations? Can J Fish Aquat Sci. 2001; 58: 30–38.

[14] Van Neer WV, Löugas L, Rijnsdorp AD. Reconstructing age distribution, season of capture and growth rate of fish from archaeological sites based on otoliths and vertebrae. Int J Osteoarcaeol. 1999; 9: 116–130.

[15] Van Neer W, Ervynck A, Bolle LJ, Millner RS, Rijnsdorp AD. Fish otoliths and their relevance to archaeology: an analysis of medieval, post-medieval, and recent material of plaice, cod and haddock from the North Sea. Environmental Archaeology. 2002; 7: 61–76.

[16] Rowell K, Dettman DL, Dietz R. Nitrogen isotopes in otoliths reconstruct ancient trophic position. Env Biol Fish. 2010; 89: 415–25.

[17] Hufthammer AK, Høie H, Folkvord A, Geffen AJ, Andersson C, Ninnemann US. Seasonality of human site occupation based on stable oxygen isotope ratios of cod otoliths. J Archaeol S. 2010; 37:78–83.

[18] Limburg KE, Walther Y, Hong B, Olson C, Storå J. Prehistoric versus modern Baltic Sea cod fisheries: selectivity across the millennia. Proc R Soc B. 2008; 275: 2659–65. doi: 10.1098/rspb.2008.0711 18755680 10.1098/rspb.2008.0711 PMC2605816

[19] Trippel EA. Age at maturity as a stress indicator in fisheries. Bioscience. 1995; 45: 759–771.

[20] Jobling M, Meløy OH, Dos Santos J, Christiansen B. The compensatory growth response of the Atlantic cod: effects of nutritional history. Aquac Int. 1994;2: 75–90.

[21] Taylor CC. Cod growth and temperature. ICES J Mar Sci. 1958; 23: 366–70.

[22] Björnsson B, Steinarsson A. The food-unlimited growth rate of Atlantic cod (Gadus morhua). Can J Fish Aquat Sci. 2002; 59: 494–502.

[23] Baumann H, Talmage SC, Gobler CJ. Reduced early life growth and survival in a fish in direct response to increased carbon dioxide. Nat Clim Change. 2012; 2: 38.

[24] Rochet MJ. Short-term effects of fishing on life history traits of fishes. ICES J Mar Sci. 1998; 55: 371–391.

[25] Bianchi G, Gislason H, Graham K, Hill L, Jin X, Koranteng K, et al Impact of fishing on size composition and diversity of demersal fish communities. ICES J Mar Sci. 2000; 57: 558–571.

[26] Andersen KH, Farnsworth KD, Thygesen UH, Beyer JE. 2007. The evolutionary pressure from fishing on size at maturation of Baltic cod. Ecol Model. 2007; 204: 246–252.

[27] Swain DP, Sinclair AF, Hanson JM. Evolutionary response to size-selective mortality in an exploited fish population. Proc R Soc B. 2007; 274: 1015–1022. doi: 10.1098/rspb.2006.0275 17264058 10.1098/rspb.2006.0275 PMC2124474

[28] Hutchings JA, Baum JK. 2005. Measuring marine fish biodiversity: temporal changes in abundance, life history and demography. Phil Trans R Soc B. 2005; 360: 315–338. doi: 10.1098/rstb.2004.1586 15814348 10.1098/rstb.2004.1586 PMC1569453

[29] Jørgensen C, Ernande B, Fiksen Ø. Size‐selective fishing gear and life history evolution in the Northeast Arctic cod. Evol Appl. 2009; 2: 356–370. doi: 10.1111/j.1752-4571.2009.00075.x 25567886 10.1111/j.1752-4571.2009.00075.x PMC3352490

[30] Cheung WW, Sarmiento JL, Dunne J, Frölicher TL, Lam VW, Palomares MD, et al Shrinking of fishes exacerbates impacts of global ocean changes on marine ecosystems. Nature Climate Change. 2013; 3:254–258.

[31] Amorosi T, McGovern TH, Perdikaris S. Bioarchaeology and cod fisheries: a new source of evidence. ICES Mar Sci Symp. 1994; 198: 31–48.

[32] Barrett JH. Fish trade in Norse Orkney and Caithness: a zooarchaeological approach. Antiquity. 1997; 71: 616–638.

[33] Perdikaris S, McGovern TH. 2009. Viking age economics and the origins of commercial cod fisheries in the North Atlantic. Beyond the catch. Fisheries of the North Atlantic, the North Sea and the Baltic, 900–1850, pp.61–90.

[34] Barrett JH, Orton D, Johnstone C, Harland J, Van Neer W, Ervynck A, et al Interpreting the expansion of sea fishing in medieval Europe using stable isotope analysis of archaeological cod bones. J Archaeol S. 2011; 38: 1516–1524.

[35] Edvardsson, R. The Role of Marine Resources in the Medieval Economy of Vestfirðir, Iceland. PhD Thesis. 2010. Graduate Center, City University of New York.

[36] Geffen AJ, Høie H, Folkvord A, Hufthammer AK, Andersson C, Ninnemann U, et al High-latitude climate variability and its effect on fisheries resources as revealed by fossil cod otoliths. ICES J Mar Sci. 2011; doi: 10.1093/icesjms/fsr017

[37] Ólafsdóttir GÁ, Westfall KM, Edvardsson R, Pálsson S. Historical DNA reveals the demographic history of Atlantic cod (Gadus morhua) in medieval and early modern Iceland. Proc R Soc Lond B. 2014; 281(1777), p.20132976. 10.1098/rspb.2013.2976 PMC3896027 24403343

[38] Barrett JH, Locker AM, Roberts CM. The origins of intensive marine fishing in medieval Europe: the English evidence. Proc R Soc Lond B. 2004; 271: 2417–2421. 10.1098/rspb.2004.2885 PMC1691892 15590590

[39] Ogilvie AE, Jónsson T. " Little ice age" research: A perspective from Iceland. Climatic Change. 2001; 48: 9–52.

[40] Patterson WP, Dietrich KA, Holmden C, Andrews JT. Two millennia of North Atlantic seasonality and implications for Norse colonies. Proc Nat Acad Sci. 2010; 107: 5306–5310. doi: 10.1073/pnas.0902522107 20212157 10.1073/pnas.0902522107 PMC2851789

[41] Drinkwater KF. The response of Atlantic cod (Gadus morhua) to future climate change. ICES J Mar Sci. 2005; 62: 1327–1337.

[42] Freitas C, Olsen EM, Moland E, Ciannelli L, Knutsen H. Behavioral responses of Atlantic cod to sea temperature changes. Ecol Evol. 2015; 5: 2070–2083. doi: 10.1002/ece3.1496 26045957 10.1002/ece3.1496 PMC4449760

[43] Dalley EL, Anderson JT. Age-dependent distribution of demersal juvenile Atlantic cod (Gadus morhua) in inshore/offshore northeast Newfoundland. Can J Fish Aquat Sci. 1997; 54:168–176.

[44] Gibb FM, Gibb IM, Wright PJ. Isolation of Atlantic cod (Gadus morhua) nursery areas. Mar Biol. 2007; 151:1185–1194.

[45] Campana SE. How reliable are growth back-calculations based on otoliths? Can J Fish Aquat Sci. 1990; 47: 2219–2227.

[46] Mirman D. Growth curve analysis and visualization using R. CRC Press.2014.

[47] Bates D, Mächler M, Bolker B, Walker S. Fitting Linear Mixed-Effects Models Using lme4. J Stat Softw. 2015; 67: 1–48. http://dx.doi.org/10.18637/jss.v067.i01

[48] R Core Team. R: A language and environment for statistical computing. 2014. http://www.R-project.org/

[49] Haddon M. Modelling and quantitative methods in fisheries, 1st edn. London, UK: Chapman and Hall, CRC Press.2001.

[50] Ogle DH. Introductory fisheries analyses with R. Boca Raton: CRC Press.2016.

[51] Ogle DH. FSA: fisheries stock analysis.R Package Version 0.8.5; 2016

[52] Jørgensen T. Long-term changes in growth of North-east Arctic cod (Gadus morhua) and some environmental influences. ICES J Mar Sci. 1992; 49: 263–278.

[53] Rätz HJ, Lloret J. Variation in fish condition between Atlantic cod (Gadus morhua) stocks, the effect on their productivity and management implications. Fish Res. 2003; 60: 369–380.

[54] Astthorsson OS, Gislason A, Gudmundsdottir A. Distribution, abundance and length of pelagic juvenile cod in Icelandic waters in relation to environmental conditions. In ICES Marine Science Symposia 1994 (Vol. 198, pp. 529–541). Copenhagen, Denmark: International Council for the Exploration of the Sea.

[55] Beaugrand G, Brander KM, Souissi JALS, Reid PC. Plankton effect on cod recruitment in the North Sea. Nature. 2003; 426: 661 doi: 10.1038/nature02164 14668864 10.1038/nature02164

[56] Kristiansen T, Drinkwater KF, Lough RG, Sundby S. Recruitment variability in North Atlantic cod and match-mismatch dynamics. PLoS One. 2011; 6(3):e17456 doi: 10.1371/journal.pone.0017456 21408215 10.1371/journal.pone.0017456 PMC3049760

[57] Brander KM. The effect of temperature on growth of Atlantic cod (Gadus morhua L.). ICES J Mar Sci. 1995; 52: 1–10.

[58] Pörtner HO, Berdal B, Blust R, Brix O, Colosimo A, De Wachter B, et al Climate induced temperature effects on growth performance, fecundity and recruitment in marine fish: developing a hypothesis for cause and effect relationships in Atlantic cod (Gadus morhua) and common eelpout (Zoarces viviparus). Cont Shelf Res. 2001; 21:1975–1997.

[59] Petersen MF, Steffensen JF. Preferred temperature of juvenile Atlantic cod Gadus morhua with different haemoglobin genotypes at normoxia and moderate hypoxia. J Exp Biol. 2003; 206: 359–364. 12477905 10.1242/jeb.00111

[60] Astthorsson OS, Gislason A, Jonsson S., Climate variability and the Icelandic marine ecosystem. Deep Sea Res Part II Top Stud Oceanogr. 2007; 54: 2456–2477.

[61] Jónsdóttir ÓD, Imsland AK, Daní AK, Marteinsdóttir G. Genetic heterogeneity and growth properties of different genotypes of Atlantic cod (Gadus morhua L.) at two spawning sites off south Iceland. Fish Res. 2002; 55: 37–47.

[62] Jónsdóttir IG, Marteinsdóttir G, Pampoulie C. Relation of growth and condition with the Pan I locus in Atlantic cod (Gadus morhua L.) around Iceland. Mar Biol. 2008; 154: 867–874.

