# Supplementary material for: A millennium of north-east Atlantic cod juvenile growth trajectories inferred from archaeological otoliths
Source: PLoS One. 2017 Oct 27;12(10):e0187134. doi: 10.1371/journal.pone.0187134 (PMC5659679; doi:10.1371/journal.pone.0187134)
Supplement: S1 Table — (DOCX) [file pone.0187134.s001.docx]

**S1 Table.** Full results from the first generalised linear mixed model with polynomial terms, examining differences in growth patterns between all archaeological deposits. Boldface denotes significant effects and bold, italic trends.

|  | Estimate | SE | t-value | p-value |
| --- | --- | --- | --- | --- |
| **Intercept** | **1.080** | **0.043** | **25.390** | **0.000** |
| **Linear term** | **0.878** | **0.022** | **40.644** | **0.000** |
| **Quadric term** | **-0.110** | **0.021** | **-5.285** | **0.000** |
| AD 971 | -0.017 | 0.057 | -0.306 | 0.759 |
| AD 1360 | 0.014 | 0.083 | 0.162 | 0.871 |
| AD 1410 | -0.024 | 0.048 | -0.502 | 0.616 |
| AD 1490 | -0.061 | 0.073 | -0.835 | 0.404 |
| AD 1570 | -0.010 | 0.068 | -0.143 | 0.886 |
| AD 1621 | 0.052 | 0.078 | 0.664 | 0.507 |
| AD 1637 | 0.049 | 0.054 | 0.914 | 0.361 |
| AD 1649 | 0.000 | 0.073 | 0.002 | 0.998 |
| AD 1660 | -0.025 | 0.064 | -0.388 | 0.698 |
| **AD 1680** | **-0.150** | **0.074** | **-2.025** | **0.043** |
| AD 1710 | -0.104 | 0.066 | -1.576 | 0.115 |
| AD 1744 | -0.018 | 0.060 | -0.300 | 0.764 |
| AD 1785 | -0.103 | 0.078 | -1.321 | 0.186 |
| AD 1795 | -0.049 | 0.059 | -0.834 | 0.404 |
| AD 1820 | 0.020 | 0.062 | 0.328 | 0.743 |
| AD 1890 | -0.090 | 0.064 | -1.413 | 0.158 |
| Linear term:AD 971 | -0.001 | 0.028 | -0.024 | 0.981 |
| Linear term:AD 1360 | -0.044 | 0.041 | -1.075 | 0.282 |
| Linear term:AD 1410 | -0.021 | 0.024 | -0.865 | 0.387 |
| Linear term:AD 1490 | -0.028 | 0.036 | -0.774 | 0.439 |
| Linear term:AD 1570 | 0.027 | 0.033 | 0.808 | 0.419 |
| **Linear term:AD 1621** | **0.122** | **0.038** | **3.220** | **0.001** |
| Linear term:AD 1637 | 0.007 | 0.026 | 0.256 | 0.798 |
| Linear term:AD 1649 | 0.016 | 0.036 | 0.460 | 0.646 |
| Linear term:AD 1660 | -0.019 | 0.031 | -0.601 | 0.548 |
| **Linear term:AD 1680** | **-0.091** | **0.036** | **-2.512** | **0.012** |
| Linear term:AD 1710 | 0.002 | 0.032 | 0.065 | 0.948 |
| Linear term:AD 1744 | -0.018 | 0.030 | -0.606 | 0.544 |
| Linear term:AD 1785 | -0.033 | 0.038 | -0.855 | 0.393 |
| Linear term:AD 1795 | 0.001 | 0.029 | 0.030 | 0.976 |
| Linear term:AD 1820 | 0.039 | 0.031 | 1.277 | 0.202 |
| Linear term:AD 1890 | -0.047 | 0.031 | -1.531 | 0.126 |
| Quadric term:AD 971 | 0.009 | 0.028 | 0.341 | 0.733 |
| ***Quadric term:AD 1360*** | ***-0.070*** | ***0.041*** | ***-1.715*** | ***0.086*** |
| Quadric term:AD 1410 | -0.003 | 0.024 | -0.109 | 0.913 |
| Quadric term:AD 1490 | -0.002 | 0.036 | -0.064 | 0.949 |
| **Quadric term:AD 1570** | **-0.070** | **0.033** | **-2.113** | **0.035** |
| Quadric term:AD 1621 | 0.006 | 0.038 | 0.164 | 0.869 |
| Quadric term:AD 1637 | 0.007 | 0.026 | 0.258 | 0.797 |
| Quadric term:AD 1649 | -0.020 | 0.036 | -0.568 | 0.570 |
| Quadric term:AD 1660 | -0.027 | 0.031 | -0.861 | 0.389 |
| Quadric term:AD 1680 | 0.010 | 0.036 | 0.276 | 0.783 |
| Quadric term:AD 1710 | -0.012 | 0.032 | -0.380 | 0.704 |
| Quadric term:AD 1744 | -0.015 | 0.029 | -0.522 | 0.602 |
| Quadric term:AD 1785 | 0.019 | 0.038 | 0.505 | 0.613 |
| Quadric term:AD 1795 | -0.027 | 0.029 | -0.952 | 0.341 |
| Quadric term:AD 1820 | 0.028 | 0.030 | 0.935 | 0.350 |
| Quadric term:AD 1890 | 0.013 | 0.031 | 0.416 | 0.678 |
