# Supplementary material for: A millennium of north-east Atlantic cod juvenile growth trajectories inferred from archaeological otoliths
Source: PLoS One. 2017 Oct 27;12(10):e0187134. doi: 10.1371/journal.pone.0187134 (PMC5659679; doi:10.1371/journal.pone.0187134)
Supplement: S2 Table — (DOCX) [file pone.0187134.s002.docx]

|  | Estimate | SE | t-value | p-value |
| --- | --- | --- | --- | --- |
| **Intercept** | 1.044 | 0.023 | 45.960 | 0.000 |
| **Linear term** | 0.873 | 0.012 | 72.251 | 0.000 |
| **Quadric term** | -0.107 | 0.012 | -9.074 | 0.000 |
| Early Modern period | 0.022 | 0.028 | 0.765 | 0.444 |
| Medieval period | 0.014 | 0.029 | 0.493 | 0.622 |
| Linear term: Early Modern | 0.006 | 0.014 | 0.429 | 0.668 |
| Linear term: Medieval | -0.012 | 0.014 | -0.863 | 0.388 |
| Quadric term: Early Modern | -0.017 | 0.014 | -1.248 | 0.212 |
| Quadric term: Medieval | -0.008 | 0.014 | -0.563 | 0.573 |

**S2 Table.** Full results from the second generalised linear mixed model with polynomial terms. examining differences in growth patterns between the Medieval. Early Modern and Modern periods. Boldface denotes significant effects and bold. italic trends.
