# Supplementary material for: A millennium of north-east Atlantic cod juvenile growth trajectories inferred from archaeological otoliths
Source: PLoS One. 2017 Oct 27;12(10):e0187134. doi: 10.1371/journal.pone.0187134 (PMC5659679; doi:10.1371/journal.pone.0187134)
Supplement: S2 File — (DOCX) [file pone.0187134.s004.docx]

R code for fitting the linear mixed model (#1) and the vBGF models (#2)

# 1. Fit a generalised linear mixed models with a second order polynomial

t <- poly(unique(data$year), 2)

# create orthogonal polynomial time variables in data frame

data[,paste("ot", 1:2, sep="")] <- t[data$year, 1:2]

# Define the model

model.full <- lmer(growth ~(ot1+ot2)*as.factor(time)+(ot1+ot2|age)+(1|ID), data=data, REML=FALSE)

# define model coefficients and estimate significance

model.coefs <- data.frame(coef(summary(model.full)))

model.coefs$p <- 2*(1-pnorm(abs(model.coefs$t.value)))

model.coefs

# 2. Estimate parameters and compare vBGF models

# Define model with different parameter values for each period

vbLKt<-growth~Linf[per]*1-exp(-K[per]*(year-t0[per]))

# Define model with same parameter values for each period

vb0<-growth~Linf*1-exp(-K*(year-t0))

# Define starting values for same parameters model

sv0<-vbStarts(growth~year, data=data)

# Define starting values for different parameters model

svLKt<-Map(rep,sv0,c(3,3,3))

# Fit same parameters model

fit0<-nls(vb0, data=data,start=sv0)

# Fit different parameters model

fitLKt<-nls(vbLKt, data=data,start=svLKt)

# Compare models

lrt(fit0, com=fitLKt)
